# Persistent Shoulder Pain Due to a Suprascapular Nerve Injury in the Setting of Trauma

**DOI:** 10.7759/cureus.4224

**Published:** 2019-03-11

**Authors:** Levonti Ohanisian, Nicholas Brown, StevenClaude D White, David Rubay, Paul M Schwartz

**Affiliations:** 1 Internal Medicine, Florida Atlantic University Charles E. Schmidt College of Medicine, Boca Raton, USA; 2 Physical Medicine and Rehabilitation, Florida Atlantic University Charles E. Schmidt College of Medicine, Boca Raton, USA; 3 Genetics, Emory University, Atlanta, USA; 4 Surgery, Florida Atlantic University Charles E. Schmidt College of Medicine, Boca Raton, USA

**Keywords:** suprascapular nerve, suprascapular, trauma, sports trauma, shoulder

## Abstract

Suprascapular neuropathy is a rare cause of shoulder pain with an injury to the nerve intrinsically related to the anatomy and course of the suprascapular nerve. The common etiologies of a suprascapular nerve injury include repetitive overhead activity, rotator cuff pathology, and compression of the nerve at either the suprascapular or the spinoglenoid notch secondary to space-occupying lesions. Although uncommon, suprascapular nerve damage has been associated with scapular fractures previously. However, there is a scarcity of literature describing a suprascapular nerve injury as the etiology of persistent shoulder pain after trauma. We present the case of a 52-year-old male who was struck by a motor vehicle, suffered a scapular fracture, and developed persistent shoulder pain secondary to a suprascapular nerve injury diagnosed 15 months post trauma.

## Introduction

Suprascapular neuropathy is a rare cause of shoulder pain [1]. The anatomy of the suprascapular nerve renders it vulnerable to dynamic and static compression and injury [1]. The course of the suprascapular nerve makes it subject to injury as it passes through the suprascapular and the spinoglenoid notch [2-3]. The common etiologies of a suprascapular nerve injury include repetitive overhead activity, rotator cuff pathology, and compression of the nerve at either the suprascapular or the spinoglenoid notch secondary to space-occupying lesions [4]. A suprascapular nerve injury presents as a vague shoulder pain with associated weakness in shoulder abduction and external rotation [3]. Later, the muscles of the shoulder girdle supplied by the suprascapular nerve may atrophy [3]. The differential diagnosis includes cervical radiculopathy, brachial plexopathy, and rotator cuff injury [3]. Diagnosis is made with a history and physical examination and confirmed with electromyography (EMG) [3]. Treatment includes early rehabilitation involving the active and passive range of motion (ROM) exercises, with the hopes of delaying muscle atrophy and preventing secondary shoulder joint pathology [3]. Although most of the literature describes suprascapular neuropathy as occurring in the setting of athletes participating in repetitive overhead movement sports, there have been reports of a suprascapular injury in the setting of trauma [3-4]. Although uncommon, suprascapular nerve damage has been associated with scapular fractures previously. However, there is a scarcity of literature describing a suprascapular nerve injury as the etiology of persistent shoulder pain after trauma. We present the case of a 52-year-old male who was struck by a motor vehicle, suffered a scapular fracture, and developed persistent shoulder pain secondary to a suprascapular nerve injury diagnosed 15 months post trauma.

## Case presentation

**Figure 1:**
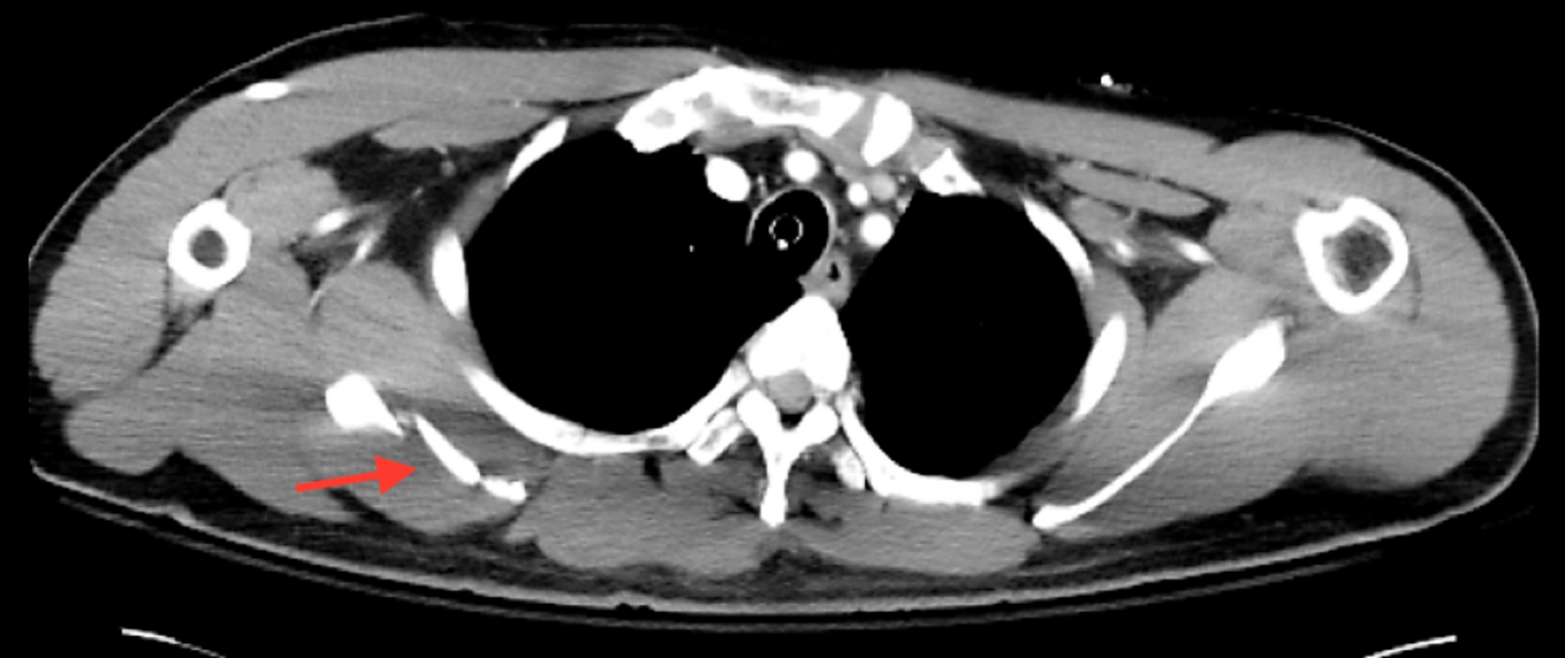
Axial CT demonstrating a right comminuted scapular fracture CT: computed tomography

**Figure 2:**
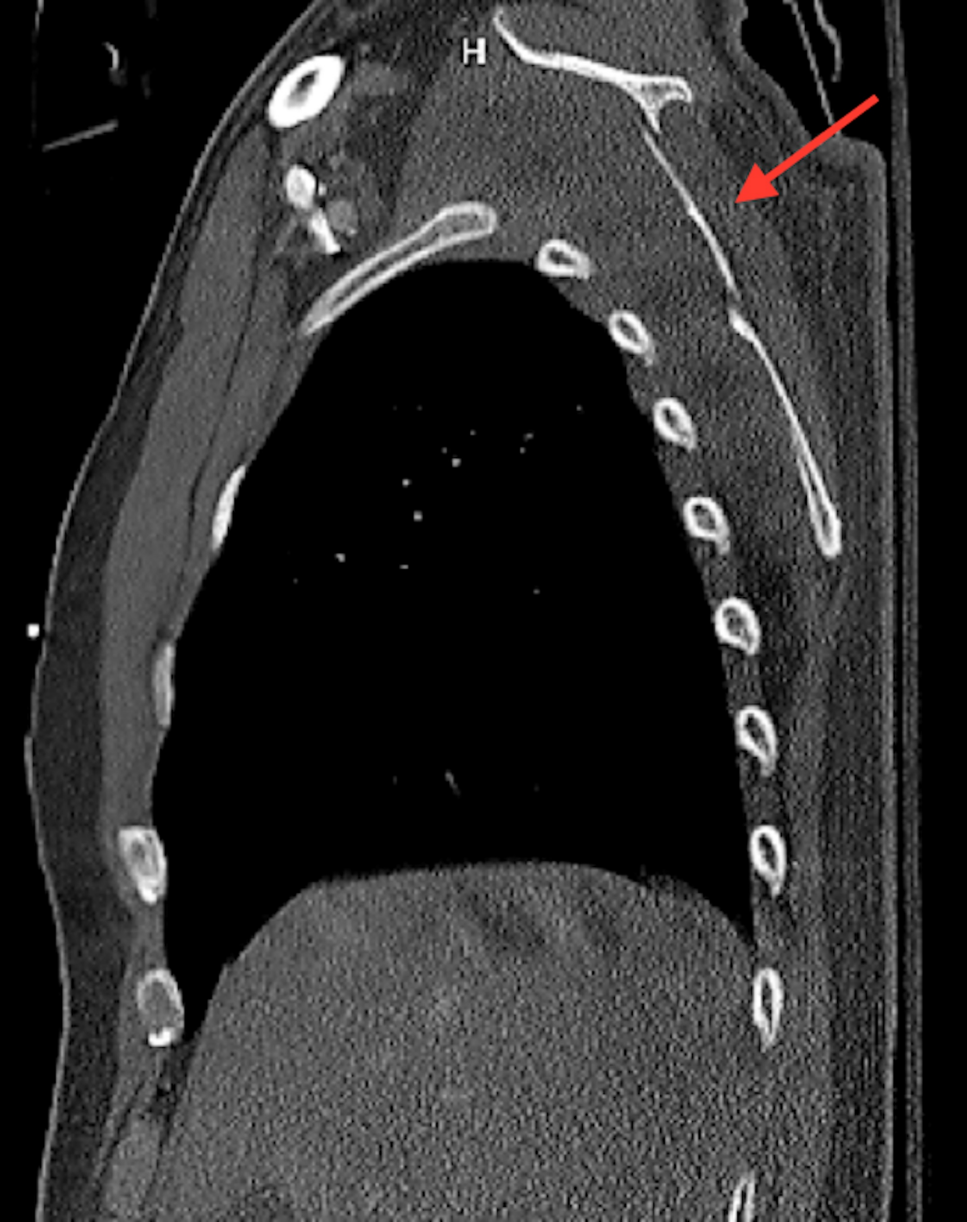
Sagittal CT demonstrating a right comminuted scapular fracture CT: computed tomography

The patient is a 52-year-old male with a history of a severe motor vehicle collision, who presented with the chief complaint of persistent right shoulder weakness. Fifteen months prior, he was struck as a pedestrian by a motor vehicle. As a result, he sustained multiple severe facial fractures, fractures of the first, third, fourth, and fifth right ribs, a fracture of the left first rib, fractures of the left T1 and T2 transverse processes, and a comminuted transverse fracture of the right scapular body (Figure 1, Figure 2).

He received multiple facial reconstruction surgeries and was kept in the hospital for over one month. Since then, he had been followed by an orthopedic surgeon and received physical therapy but the right shoulder weakness persisted. Physical examination of the right shoulder in our clinic revealed marked atrophy of the infraspinatus muscle when compared to the left shoulder. In addition, there was a significant loss of range of active and passive motion in all planes of the right shoulder. EMG was performed, which demonstrated a normal insertional activity and interference pattern in the biceps and deltoid muscles, suggesting no denervation at these muscles. He had reduced recruitment, a reduced interference pattern, and a few positive sharp waves in the supraspinatus muscle, suggesting denervation. The infraspinatus muscle had little to no activity and was difficult to measure due to significant atrophy. These findings suggested that there was an injury to the suprascapular nerve with more severe denervation at the branch to the infraspinatus muscle. The suspected cause of the injury was the scapular fracture. The patient was sent for physical therapy and was unwilling to consider further invasive treatment at this time. Although the patient's shoulder pain was stable at the time, it was thought that physical therapy could be useful to prevent further atrophy and weakness.

## Discussion

**Figure 3:**
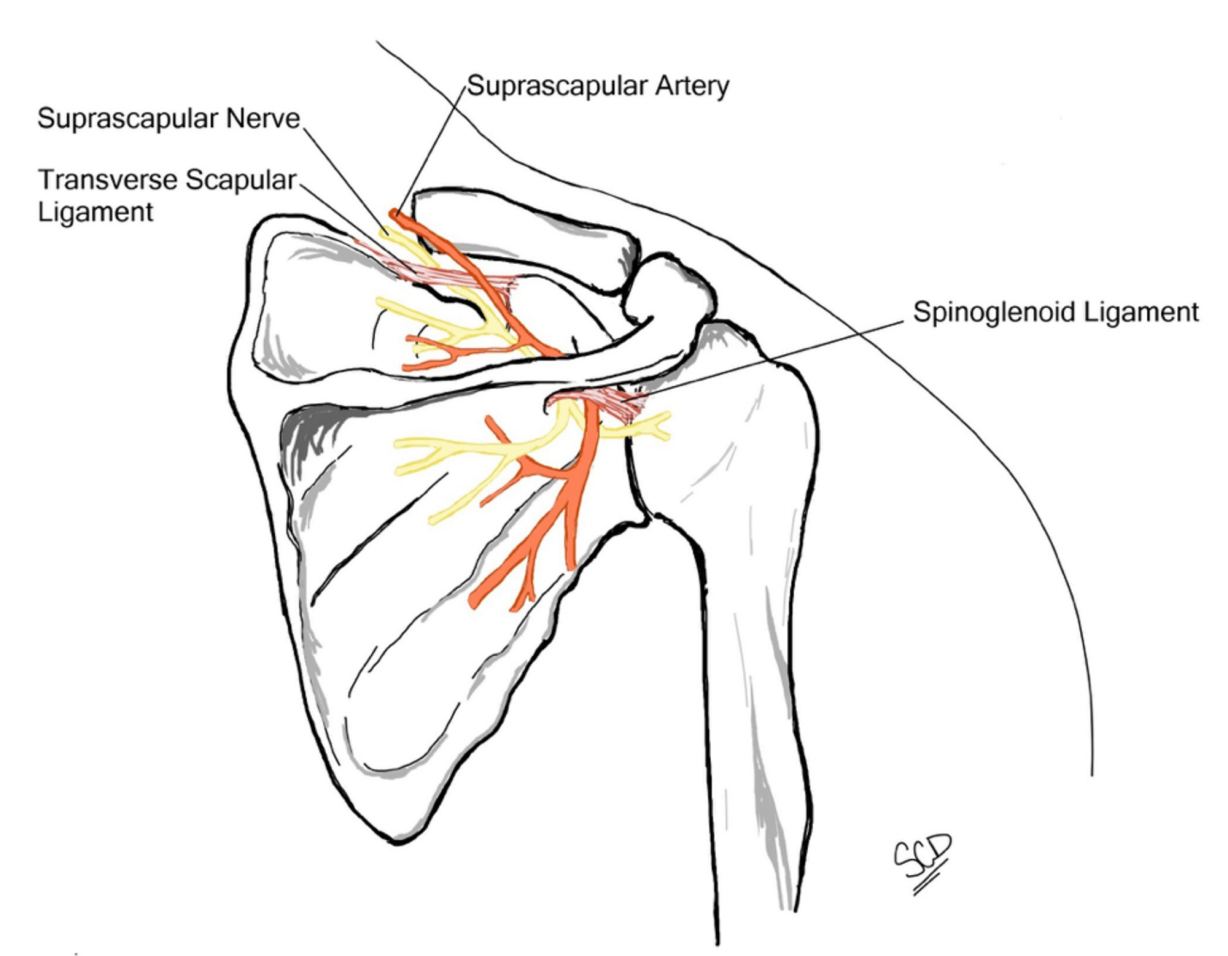
Illustration depicting the suprascapular nerve course relative to scapular ligamentous structures

A complete understanding of the relevant anatomy, including the shoulder joint and brachial plexus anatomy, is essential in comprehending the pathophysiology involved in suprascapular neuropathy. The suprascapular nerve has its main originating contributions from the upper part of the brachial plexus, more specifically from the C5 and C6 vertebral rami, with variable contributions from the fourth cervical ramus [1]. In a study using 37 cadavers, Shin et al. reported that the suprascapular nerve involved the ventral rami of the C5 and C6 roots in 76% of the samples, C4, C5, and C6 in 18%, and only the C5 root in 6% [5]. After its exit from the brachial plexus, the suprascapular nerve runs laterally through the posterior cervical triangle posterior to the clavicle and across the superior border of the scapula, finding its way into the suprascapular notch [6]. The ligamentous anatomy surrounding the suprascapular notch illustrates areas where the suprascapular nerve may be affected (Figure 3).

For instance, if the transverse scapular ligament superiorly ossifies or hypertrophies, there may be resulting stenosis and compression of the nerve. After the nerve has passed through the suprascapular notch, it courses posterolaterally through the supraspinatus fossa. During this time, it provides motor branches to the supraspinatus muscle while receiving sensory input [6-7] and continues posterolaterally, entering the spinoglenoid notch to innervate the infraspinatus muscle [1]. The spinoglenoid notch is bordered superiorly by the spinoglenoid ligament (inferior transverse scapular ligament) [1,6-7]. The spinoglenoid ligament’s insertion into the posterior capsule creates an area of possible injury to the suprascapular nerve. Specifically, Liveson et al. have reported three cases of suprascapular nerve injuries at the spinoglenoid notch [2].

The diagnosis of a nerve injury can be made with a complete history and physical examination. These findings can be confirmed with EMG and nerve conduction studies [3-4]. Treatment is initially nonoperative and early rehabilitation involving active and passive ROM exercises with the hopes of delaying muscle atrophy and preventing secondary shoulder joint pathology [3]. Pain may be managed with the use of non-steroidal anti-inflammatory drugs (NSAIDs) and, in the setting of symptomatic progression, surgical intervention with either the open or the arthroscopic technique [4]. The advent of arthroscopic techniques has increased the number of suprascapular nerve releases performed by orthopedic surgeons [1].

Much of the literature describes suprascapular lesions in the setting of athletic injuries involved in repetitive overhead motion sports [4]. However, there have been few reports of suprascapular nerve damage in the setting of trauma. Yoon et al. reported four cases of suprascapular nerve injuries in the setting of trauma, two of which included patients who were involved in motor vehicle accidents [3]. Scapular fractures approach the rate of distal tibia and humerus fractures with a rate of 0.7% of all fractures [8]. Suprascapular nerve damage has been associated with scapular fractures previously, although this is uncommon. Herrera et al. studied 22 patients who suffered scapular fractures in the setting of trauma, seven of which developed suprascapular nerve injury [9]. However, there is a scarcity of literature describing suprascapular nerve injuries as the etiology of persistent shoulder pain after trauma.

Suprascapular neuropathy has become a much more frequent diagnosis in recent years. A meta-analysis that investigated cases of suprascapular neuropathy between 1959 and 2001 revealed 88 reported cases [10]. Since the number of cases has dramatically increased, likely due to awareness of suprascapular nerve pathology in athletes that perform overhead motions [4]. However, it is important to acknowledge that other settings can be considered high risk for suprascapular nerve injuries. One of these circumstances is trauma. Our case reports a 52-year-old male who presented with persistent suprascapular neuropathy 15 months after being struck by a motor vehicle. Shoulder pain in the setting of trauma, particularly with coexisting evidence of scapular fracture, should include suprascapular neuropathy within the differential diagnosis. Additionally, patients with long-term shoulder pain after trauma should be worked up for a suprascapular nerve injury. Subsequent targeted history and physical examination, as well as EMG and nerve conduction studies, should be used to assess for this pathology.

## Conclusions

Although rare, suprascapular neuropathy has become a more common diagnosis in recent years. The suprascapular nerve is susceptible to damage due to its course through the transverse scapular ligament and spinoglenoid ligament. Despite most of the literature describing suprascapular nerve damage due to sports-related injuries and repetitive movement, trauma has also been reported as a cause of injury. Specifically, a scapular fracture has proven to be an uncommon but known cause of suprascapular nerve injury. Our paper depicts a unique case where persistent suprascapular neuropathy was diagnosed using EMG 15 months after trauma. In conclusion, our case demonstrates that in patients with a history of trauma, a suprascapular nerve injury should be placed on the differential diagnosis of persistent shoulder pain.
